# Reduced perioperative sensory impairment could lower postoperative delirium incidence: a before-and-after study in older patients with hip fracture

**DOI:** 10.1186/s12877-025-06318-5

**Published:** 2025-08-30

**Authors:** Jan Wilhelm Busse, Alexander Ranker, Manfred Gogol, Christian Macke, Emmanouil Liodakis, Derya Strack, Lukas Hinken, Carolin Jung

**Affiliations:** 1 Department of Anesthesiology and Intensive Care Medicine, Hannover Medical School, Carl-Neuberg-Straße 1, Hannover, 30625 Germany; 2 Department for Rehabilitation and Sports Medicine, Hannover Medical School, Carl-Neuberg-Straße 1, Hannover, 30625 Germany; 3 Department of Trauma Surgery, Hannover Medical School, Carl-Neuberg-Straße 1, Hannover, 30625 Germany

**Keywords:** Postoperative delirium, Emergence delirium, Sensory aid, Perioperative care, Hip fracture, Prevention, Geriatric assessment, 3D-CAM, QoR-9

## Abstract

**Background:**

Postoperative delirium (POD) is a common complication in older patients. Multicomponent prevention reduces POD but requires substantial resources. The effect of single interventions derived from multicomponent programs remains elusive, but it is essential to assess their effect to identify which components are meaningful. This study investigates whether a single intervention from a multicomponent program may reduce the incidence of POD.

**Methods:**

This prospective, single-center before-and-after study evaluated older surgery patients at a university hospital’s geriatric trauma center between July 2020 and November 2021. Inclusion criteria were age ≥ 70 years and a proximal femoral fracture. A control cohort (CC) left their dentures, vision, and hearing aids on the ward throughout surgery, while an intervention cohort (IC) used them until anesthesia induction and received them back when they regained consciousness. Therefore, IC obtained a transport case for hygienic storage and perioperative device management. The time of sensory aid removal was measured to ensure proper implementation. Outcomes were the incidence of POD (assessed via the 3-min Diagnostic Confusion Assessment Method at admission and twice daily for three days postoperatively), patient satisfaction, and recovery (evaluated via Quality of Recovery-9), length of hospital stay, postoperative monitoring time, Barthel-index, and 30-day mortality.

**Results:**

248 patients were screened for eligibility. Following a dropout rate of *n* = 166, the cohorts were: CC *n* = 40 and IC *n* = 42. Both cohorts had similar baseline and treatment characteristics. The intervention significantly reduced the median time of sensory aid removal (CC 12h 14min, IC 12min, *p* < 0.001), and the incidence of POD overall (CC 65.0%, IC 40.5%, *p* = 0.026). The length of hospital stay and long-term recovery outcomes remained insignificant. However, postoperative monitoring time correlated with time of sensory aid removal (r_s_ = 0.518, *p* < 0.001, *n* = 62), and median quality of recovery improved on average (CC 12.75, IC 15.62, *p* < 0.001).

**Conclusion:**

Reduced perioperative sensory impairment may reduce the incidence of POD, decrease postoperative monitoring time, and be associated with a better postoperative recovery. Therefore, multimodal preventative efforts should include reducing sensory aid removal.

**Trial registration:**

German Clinical Trial Register (DRKS-ID DRKS00022085); 07/07/2020

## Background

Postoperative delirium (POD) is a serious complication of surgery and anesthesia and a common challenge in geriatric trauma patients [1, 2]. It is defined as the emergence of delirium within one week following surgery [3]. In general, delirium refers to a sudden deterioration in awareness, attention, and orientation that cannot be explained by a neurocognitive disorder or a medical condition [4]. The etiology of delirium remains elusive; however, it appears to be a multicausal interrelationship between risk factors and triggering events [1].

Elderly patients with hip fractures are at high risk of POD [5]. They accumulate multiple predisposing factors (e.g., cognitive and functional impairment, sensory impairment, multiple comorbidities, advanced age, malnutrition) and are exposed to several precipitating events (e.g., hip fracture, urgent and trauma admission, use of bladder catheterization) [6, 7]. Thus, POD is the most common complication in this entity, with up to 53.3% of patients being affected [5].

The repercussions and long-term outcomes of a POD are severe. Especially older patients are at risk of long-term cognitive impairment, increased mortality, and morbidity [8, 9]. The financial burden of POD is staggering, with estimates placing the annual damage of hyperactive delirium alone at approximately 1 million euros (€) per ward [10]. These expenditures are expected to rise in the future because of an aging population and a corresponding estimated increase in hip fractures [11, 12].

Therefore, effective POD prevention is necessary and appropriate [13, 14]. Numerous studies and reviews have shown the effectiveness of multicomponent nonpharmacological prevention programs [15, 16]. Compared to multicomponent prevention programs, studies examining the implementation of a single preventative measure are rare [16]. The aim of this study is to investigate the effect of reduced perioperative sensory impairment on the prevention of POD, to assess its value in multicomponent prevention concepts.

Sensory impairment is an important risk factor for POD, and sensory deficiency is a frequent issue in geriatric individuals [1, 17, 18]. Almost every older patient requires glasses, and one in five patients depends on hearing aids [19]. The extended application of patient-owned and patient-adapted aid devices to minimize perioperative sensory aid removal could therefore be an inexpensive and effective prevention of POD. This study investigates the impact of sensory aid removal on POD as well as postoperative recovery in the elderly.

## Methods

### Design and participants

For this prospective cohort study, all patients aged ≥ 70 years who received surgery at a geriatric trauma center (level I trauma center) in Germany due to a proximal femoral fracture between July 2020 and November 2021 were screened for inclusion. All eligible patients were identified via the hospital information system. Criteria for exclusion were: (1) no sensory aid devices at hospital admission; (2) preexisting dementia; (3) perioperative stroke; (4) language barrier; and (5) preoperative delirium.

Patients were divided into a control and an intervention cohort via date of inclusion. The control cohort (CC) (July 23, 2020 – December 10, 2020) received perioperative care according to the standard care of the department. Participants in the intervention cohort (IC) received identical surgical care complemented by an intervention to reduce the time of perioperative sensory impairment (December 11, 2020 – November 12, 2021).

**Fig. 1:**
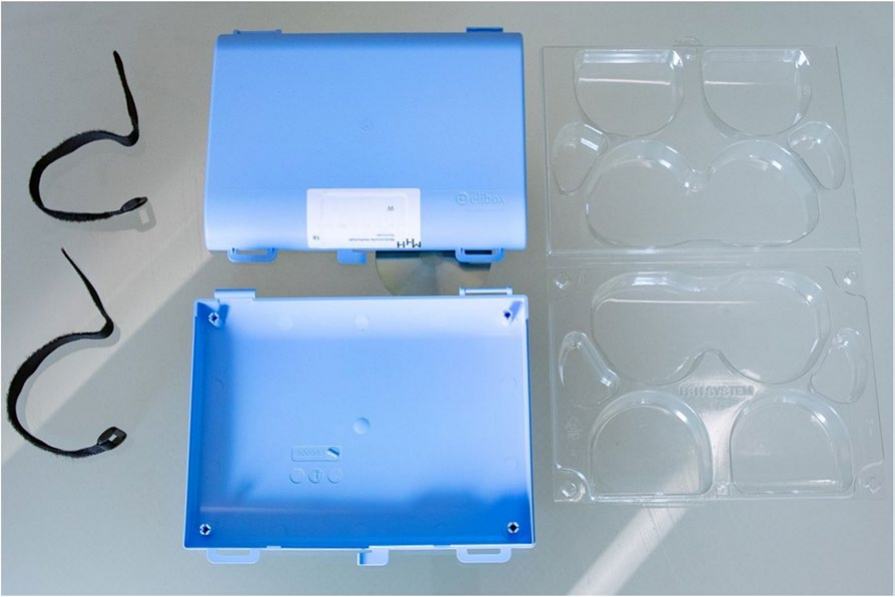
Transport case used to store sensory aid devices perioperatively. The blue outer casing consists of two parts (Center; material: acrylonitrile–butadiene–styrene), a transparent inlay (Right; material: amorphous polyethylene terephthalate) with preformed recesses for SAD (dentures, glasses, and hearing aids), and two black hook-and-loop fasteners (Left; material: polyamide) for mounting purposes. The outer casing and mounting straps are reusable after disinfection, the inlay is a one-way and person-related item. (Photo credit: Karin Kaiser/ Hannover Medical School)

Patients in the IC were equipped with a transport case (Elibox®, H + H SYSTEM GmbH, Austria) perioperatively, which allowed the hygienic storage of their own and personalized sensory aid devices (SAD) (dentures, glasses, and hearing aids) in the operating theater and during postoperative care (Fig. 1). The storage box was mounted via hook-and-loop fasteners to the patient’s bed on admission until the patient was discharged. Thus, patients were able to keep their SAD with them until anesthetic induction and regain them postoperatively in the recovery room or in the intensive care unit (ICU) as soon as they were able to manage them again. Patients in the historical control had to leave their SAD at the ward to prevent them from being lost or damaged during the perioperative procedure, as it was standard of care until then. They regained their SAD after returning to the ward from the post-anesthesia care unit (PACU) or ICU, or on the ICU if aid devices were irregularly fetched from the general ward.

The new perioperative procedure regarding SAD was implemented on December 11, 2020. No other changes in treatment were made.

### Assessment of baseline and treatment characteristics

Informed consent was obtained in advance from all study participants. Baseline data, including sex, age at the time of surgery, and fracture type (femoral neck, intertrochanteric, and subtrochanteric), were registered. Anesthesia records supplemented additional information on the American Society of Anesthesiologists (ASA)—classification (2, 3, or 4), number of risk factors for postoperative nausea and vomiting (PONV) and metabolic equivalent of task (MET) [20–22]. The preoperative interview with patients included the assessment of required SAD (hearing aids needed [one-sided vs. two-sided], glasses needed, dentures needed [partial vs. full]) and whether SAD had been taken with them on hospital admission (all, only dentures, only hearing aids, partial), as well as the last oral intake to calculate fasting time before surgery. The prevalence of cognitive impairment was assessed via the Montreal Cognitive Assessment (MoCA) [23, 24]. 3-Minute Diagnostic Interview for Confusion Assessment Method-defined delirium (3D-CAM) was used to assess the prevalence of preoperative delirium as part of this research [25, 26]. Admission and discharge reports provided the Parker Mobility Score, the Identification of Seniors at Risk Score, and the information necessary to evaluate comorbidity using the Charlson Comorbidity Index [27–30]. Concentrations of hemoglobin, creatinine, and c-reactive protein were determined from routine admission blood samples.

Additional variables were surgical treatment (endoprosthetic surgery, osteosynthesis), type of postoperative monitoring (PACU vs. ICU), time of postoperative monitoring (PACU vs. ICU), time to surgery, surgery time, and length of hospital stay. In addition, anesthetic technique (general anesthesia vs. spinal anesthesia) was obtained from anesthesia documentation.

Perioperative sensory impairment was defined as the time of consciousness without SAD and was calculated using time stamps from electronic health records (calculation: sensory impairment = [end of sensory impairment – start of sensory impairment] – [time of intubation]). The start and end of sensory impairment (removal and application of SAD) were defined based on the historical standard operating procedures for CC (start of sensory aid removal = time of transfer to the operating room; end of sensory aid removal = time of transfer to the normal ward) and according to the study protocol for IC (start of sensory aid removal = start of preoxygenation; end of sensory aid removal = end of anesthesia). Protocol adherence was continuously reviewed, and whenever deviations from the research protocol were identified during follow-up (e.g., SAD were spontaneously fetched on the ICU in CC or SAD were applied later than defined by the study protocol in IC), time stamps for calculating the duration of sensory impairment were corrected to ensure accurate data input and to verify a successful intervention implementation.

### Outcome measurement

All patients were monitored for POD immediately after surgery and twice a day for the following three days. The 3D-CAM score was used for POD screening [25, 26]. Participants were asked to rate their subjective recovery and level of satisfaction using the validated Quality-of-Recovery-9 score (QoR-9) [31, 32].

After 30 days, a follow-up was performed by telephone or, if feasible, in the outpatient department of the clinic. Patients or their family members were interviewed using the QoR-9 score and Barthel Index [31–33]. In addition, we evaluated mortality at follow-up.

### Study size and potential bias management

The number of participants for each cohort was based on the number of included patients who received surgery during the first six months (July 2020 – December 2020) of the intended one-year observational period. As the pandemic surge put further strain on the German healthcare system, the inclusion of patients became more difficult. The IC observational period has been extended until November 2021 to ensure equivalent sample sizes for statistical analyses.

To avoid potential distortions related to selection bias, a comprehensive screening of potentially eligible patients in the emergency room was done. Strict inclusion and exclusion criteria were applied to achieve maximum congruence across both cohorts and additional data was not recorded until inclusion. By utilizing validated assessment tools and extensive training of a single assessor by experienced physicians, an information bias attributable to inconsistent data aggregation was addressed. In order to prevent observer-expectation bias and to ensure an accurate intervention effect, the examiner did not engage in the patient's treatment. A trial registration was used to avoid selective reporting bias.

### Statistical analyses

Statistical analyses were calculated using SPSS (SPSS 28.0.1.1, SPSS Inc., Chicago, IL, USA). Normal distribution of variables was tested via the Shapiro–Wilk test and a visual examination of the histogram. Continuous data are presented as a median with an interquartile range or mean ± standard deviation, depending on distribution characteristics. Categorial variables are presented as percentages and numbers. Statistical analysis was performed with the Mann–Whitney-U test for non-parametric data, the Student’s t-test for normally distributed continuous data, and the Chi^2^-test or Fisher’s exact test for categorical variables. Spearman’s rank-order correlation was performed to analyze correlations between variables. If a value was missing, the patient was excluded for the respective analyses. A *p*-value < 0.05 was considered statistically significant, and a *p*-value < 0.001 was deemed highly significant.

## Results

**Fig. 2:**
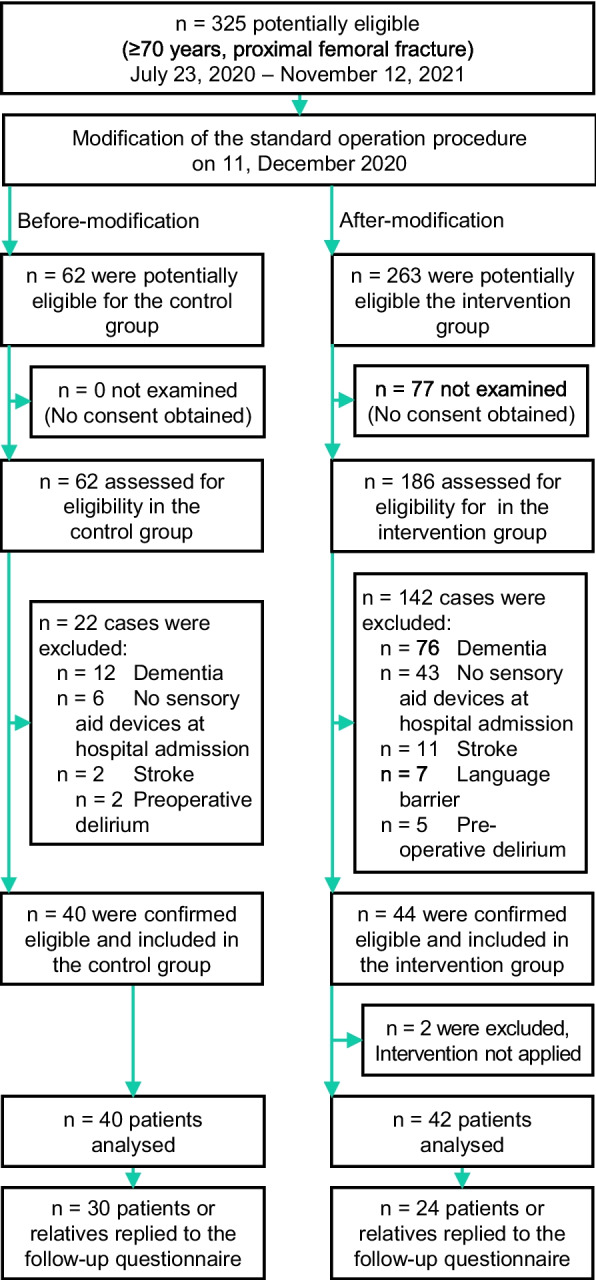
Flow chart of patient’s enrolment

Throughout the course of the study, *n* = 325 patients aged ≥ 70 years received surgery at our clinic following a proximal femoral fracture. *N* = 248 patients were assessed for eligibility (*n* = 77 could not be examined, due to missing informed consent), and *n* = 164 of those cases revealed exclusion criteria (dementia *n* = 88, presentation of patient without SAD at hospital admission *n* = 49, stroke *n* = 13, language barrier *n* = 7, preoperative delirium *n* = 7); thus, *n* = 84 patients were included. *N* = 40 patients received standard care, and *n* = 42 patients obtained the intervention. Two cases accidentally received standard care and thus had to be excluded from the intervention cohort. Follow-up questionnaires were answered by 75.0% (*n* = 30) of patients or their relatives in CC versus 57.1% (*n* = 24) in IC (Fig. 2).

### Baseline and treatment characteristics

Both cohorts exhibit similar baseline characteristics (Table 1). Two-thirds of the patients were female (CC 65.0%, IC 61.9%), with a mean age of around 83 years (CC 83.80 y ± 6.25 y, IC 82.64 y ± 7.12 y), and presenting with a femoral neck fracture in every second case (CC 52.5%, IC 54.8%). In median, both groups had mild cognitive impairment at hospital admission (MoCA median: CC = 18, IC = 18) and required comparable SAD. Nearly every patient needed glasses (CC 87.5%, IC 90.5%), two-thirds required dentures (CC 70.0%, IC 64.3%), and one in five needed hearing aids (CC 20.0%, IC 28.5%). Both cohorts carried comparable SAD at the time of admission (all SAD: CC 70.0%, IC 71.4%). CC was notably less physically resilient (MET median: CC 2, IC 4), but not considerably more severely ill (ASA-Classification, Charlson Comorbidity Index), nor with more PONV-risk factors. In contrast, median preoperative fasting time was increased for the IC (CC 23 h 29 min, IC 27 h 24 min). CC was less mobile (Parker Mobility Score) but did not have a greater amount of geriatric risk factors than IC (Identification of Seniors at Risk Score). Concentrations of creatinine, c-reactive protein and in admission blood samples did not differ either.

**Table 1:** Baseline characteristics of the control and intervention cohort

Characteristic	Control	Intervention
Women	65.0% (26)	61.9% (26)
Age, years; mean (Standard deviation)	83.80 (± 6.25)	82.64 (± 7.12)
Type of fracture		
- Femoral neck	52.5% (21)	54.8% (23)
- Intertrochanteric	42.5% (17)	38.1% (16)
- Subtrochanteric	5.0% (2)	7.1% (3)
Aid devices needed		
- Hearing aids needed	20.0% (8)	28.6% (12)
•one-sided	2.5% (1)	0.0% (0)
•two-sided	17.5% (7)	28.6% (12)
- Glasses needed	87.5% (35)	90.5% (38)
- Dentures needed	70.0% (28)	64.3% (27)
•partial	22.5% (9)	7.1% (3)
•full	47.5% (19)	57.1% (24)
Devices upon admission		
- All	70.0% (28)	71.4% (30)
- Only dentures	27.5% (11)	19.0% (8)
- Only hearing aids	2.5% (1)	2.4% (1)
- Partial	0.0% (0)	7.1% (3)
ASA-classification		
- 2	35.0% (14)	45.2% (19)
- 3	57.5% (23)	50.0% (21)
- 4	7.5% (3)	4.8% (2)
PONV risk factors	3 (2 – 3)	3 (2 – 3)
MET	2 (2 – 4)	4 (2 – 5)
Fasting time, hours:minutes	23:29 (16:14 – 40:13)	27:24 (23:53 – 47:24)
Preoperative MoCA	18 (15 – 23)	18 (14.4 – 22)
Charlson Comorbidity Index	5 (4 – 6)	5 (4 – 7)
Parker Mobility Score	6 (4 – 9)	9 (6 – 9)
Identification of Seniors at risk-Score	1 (0 – 2)	1 (1 – 2)
C-reactive protein, mg/L	3.20 (1.50 – 7.05)	6.80 (1.60 – 31.20)
Creatinine, µmol/L	88.5 (71.5 – 111.5)	82.5 (69.0 – 131.0)
Hemoglobin, g/dL; mean (Standard deviation)	12.35 (± 1.69)	11.96 (± 1.89)

All values are median (interquartile range) or percentage of patients (n), unless otherwise noted

Control cohort *n* = 40; Treatment cohort *n* = 42

*Abbreviations*: *ASA* American Society of Anesthesiologists, *PONV* Postoperative nausea and vomiting, *MET* Metabolic equivalents of task, *MoCA* Montreal cognitive assessment

**Fig. 3:**
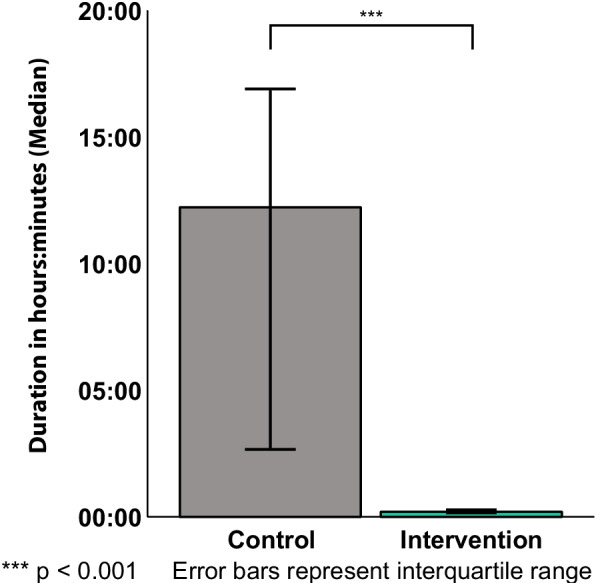
Perioperative sensory impairment (*n* = 66). Perioperative sensory impairment is defined as the time of consciousness without sensory aid devices for the control cohort and the intervention cohort (median; error bars represent the interquartile range). Calculation was performed using time stamps from electronic health records (calculation: sensory impairment = [end of sensory aid removal – start of sensory aid removal] – [time of intubation]). Protocol adherence was continuously reviewed, and time stamps for calculating the duration of sensory impairment were corrected to ensure accurate data input whenever deviations from the research protocol were identified during follow-up. Between the two cohorts, perioperative sensory impairment varied very significantly (*p* < 0.001)

There were no substantial differences in anesthesiologic or surgical procedures between groups (Table 2). Endoprosthetic surgery and general anesthesia had comparable proportions. The time from admission to surgery (CC 21h 26min, IC 22h 55min) and incision-suture time were not different. Postoperative stay (PACU vs. ICU: CC 45.0% vs. 55.0%, IC 64.3% vs. 35.7%) and duration of postoperative monitored care on ICU or PACU did not differ between groups. The time of sensory impairment was considerably reduced (median time: CC 12h 14min, IC 12min, *p* < 0.001), indicating that the intervention was successfully implemented (Fig. 3).

**Table 2:** Treatment characteristics of the control and intervention cohort

Characteristic	Control (*n* =)	Intervention (*n* =)	*p*-value
Surgical treatment			
- Endoprosthetic surgery	45.0% (18)	47.6% (20)	
- Osteosynthesis	55.0% (22)	52.4% (22)	
Anesthetic procedure			
- General anesthesia	85.0% (34)	92.9% (39)	
- Spinal anesthesia	15.0% (6)	7.1% (3)	
Postoperative care			
- PACU	45.0% (18)	64.3% (27)	
- ICU	55.0% (22)	35.7% (15)	
Postoperative monitoring time, hours:minutes			
- PACU	1:26 (0:56 – 1:38)	1:48 (0:56 – 2:29)	
- ICU	19:30 (14:04 – 44:12)	14:39 (10:19 – 53:10)	
Time to surgery, hours:minutes	21:26 (11:50 – 31:23)	22:55 (15:57 – 29:00)	
Surgery time, hours:minutes	1:05 (0:45 – 1:22)	1:07 (0:52 – 1:31)	
Sensory impairment, hours:minutes	12:14 (2:34 – 17:36)	0:12 (0:08 – 1:17)	< 0.001^***,^^†^

All values are median (interquartile range) or percentage of patients (n), unless otherwise noted

Control cohort *n* = 40; Treatment cohort *n* = 42

*Abbreviations*: *ICU* Intensive care unit, *PACU* Post anesthesia care unit

^***^ Highly significant (*p* < 0.001), †Mann–Whitney-U-Test

### Outcome characteristics

**Fig. 4:**
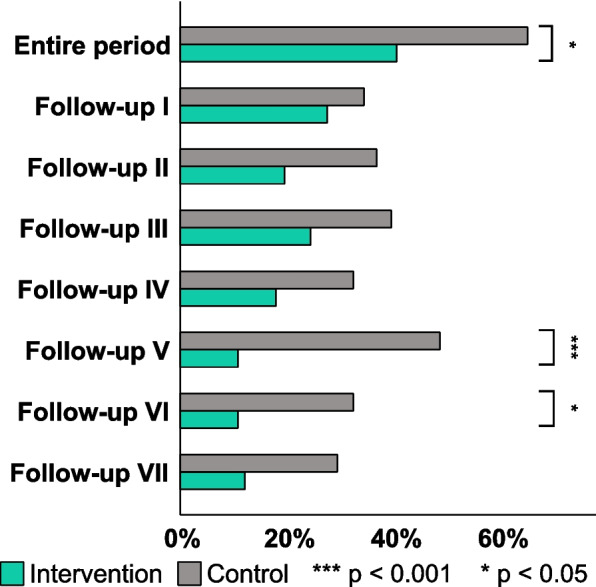
3D-CAM-Score. Postoperative delirium incidence was assessed via the 3-Minute Diagnostic interview for Confusion Assessment Method-defined Delirium (3D-CAM) during the whole observation period and at each follow-up. The incidence of postoperative delirium varied significantly during the observation (*p* = 0.026), on follow-up V (*p* < 0.001), and on follow-up VI (*p* = 0.041)

**Fig. 5:**
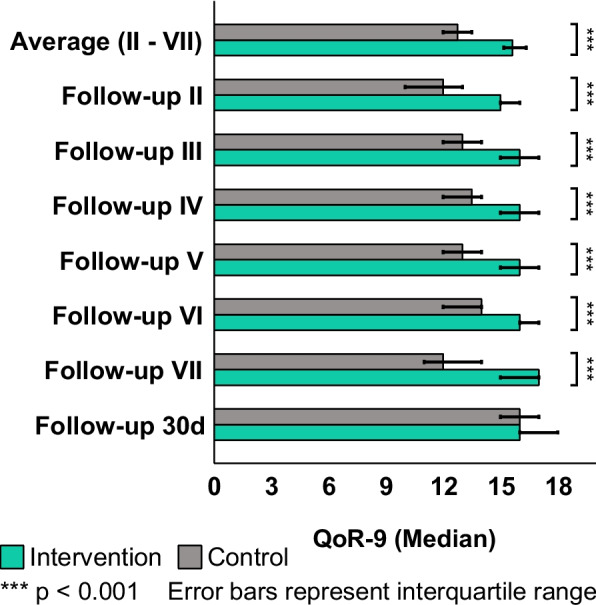
Quality-of-recovery 9. Postoperative subjective patient recovery is evaluated via Quality-of-Recovery 9 (median; error bars represent the interquartile range) for each follow-up and average for the in-hospital observation period (average II – VII). Throughout the whole observation period and at each in-hospital follow-up, the subjective patient recovery varied significantly (*p* < 0.001)

**Fig. 6:**
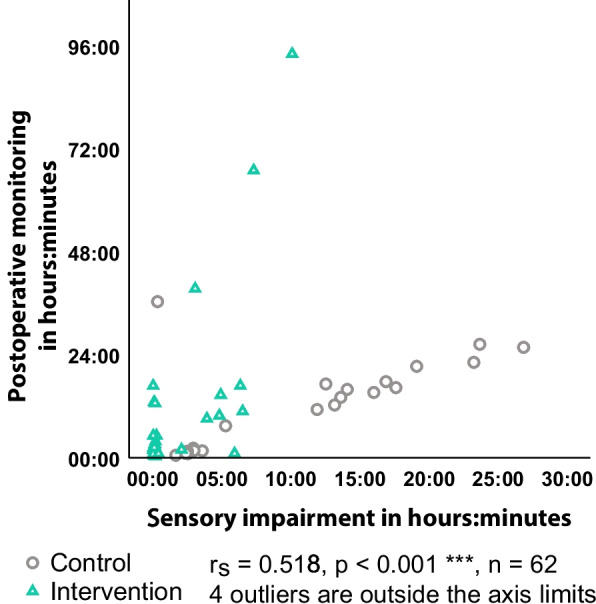
Sensory impairment – Time of postoperative monitoring. Postoperative monitoring time compared to sensory impairment. Perioperative time without sensory aid devices correlated significantly with the duration of postoperative care (rs = 0.518, *p* < 0.001, *n* = 62). To enhance readability, four outliers have been removed

Overall incidence of POD was significantly lower in the IC (CC: 65.0%, IC: 40.5%, *p* = 0.026), especially in later follow-ups (Follow-up V: CC: 48.6%, IC: 10.8%, *p* < 0.001). A visual examination of the histogram revealed a decreased POD incidence in the IC at any time (Fig. 4). Functional recovery after 30 days was unaffected by the intervention (Barthel-index median: *p* = 0.944), as were 30-day mortality (*p* = 0.486) and length of hospital stay (*p* = 0.126) (Table 3). Subjective quality of recovery and patient satisfaction were higher in IC than in CC through the entire length of hospital stay (QoR-9 average: CC 12.75, IC 15.62, *p* < 0.001) (Fig. 5). At 30-day follow-up, this difference was evened out (QoR-9: *p* = 0.135). A review of the data revealed a link between time of sensory impairment and duration of postoperative care (r_s_ = 0.518, *p* < 0.001, *n* = 62) (Fig. 6). A longer perioperative period of sensory impairment was associated with a longer stay in the PACU or ICU for postoperative treatment and observation.

**Table 3:** Outcome characteristics of the control and intervention cohort

Outcome	Control (*n* =)	Intervention (*n* =)	*p*-value
Delirium positive			
- During observation	65.0% (26/40)	40.5% (17/42)	0.026^*,^^□^
- Follow-up I	34.4% (11/32)	27.5% (11/40)	0.529^□^
- Follow-up II	36.8% (14/38)	19.5% (8/41)	0.086^□^
- Follow-up III	39.5% (15/38)	24.4% (10/41)	0.150^□^
- Follow-up IV	32.4% (12/37)	17.9% (7/39)	0.145^□^
- Follow-up V	48.6% (18/37)	10.8% (4/37)	< 0.001^***,■^
- Follow-up VI	32.4% (11/34)	10.8% (4/37)	0.041^*,^^■^
- Follow-up VII	29.4% (10/34)	12.1% (4/33)	0.132^■^
Quality-of-recovery			
- Average II-VII	12.75 (11.75 – 13.9)	15.62 (14.83 – 16.33)	< 0.001^***,^^†^
- Follow-up II	12 (9 – 14)	15 (14 – 17)	< 0.001^***,^^†^
- Follow-up III	13 (11 – 15)	16 (15 – 17)	< 0.001^***,^^†^
- Follow-up IV	13,5 (11.5 – 15)	16 (15 – 17)	< 0.001^***,^^†^
- Follow-up V	13 (11 – 14)	16 (15 – 17)	< 0.001^***,^^†^
- Follow-up VI	14 (12 – 15)	16 (15 – 17)	< 0.001^***,^^†^
- Follow-up VII	12 (11 – 14)	17 (15 – 17)	< 0.001^***,^^†^
- Follow-up 30d	16 (15 – 17)	16 (16 – 18)	0.135^†^
Length of hospital stay, days	7.748 (5.728 – 8.915)	8.278 (6.522 – 11.076)	0.126^†^
30d Barthel-Index	75 (60 – 80)	72.5 (50 – 90)	0.944^†^
30d Mortality	13.8% (4/29)	21.7% (5/23)	0.486^■^

All values are median (interquartile range) or percentage of participated patients (n/participated n)), unless otherwise noted

Control cohort *n* = 40; Treatment cohort *n* = 42

^†^Mann–Whitney-U-Test, ^□^Pearson-Chi^2^-Test, ^■^Fisher’s Exact Test

^*^ Significant (*p* < 0.05) *** Highly significant (*p* < 0.001)

## Discussion

**Fig. 7:**
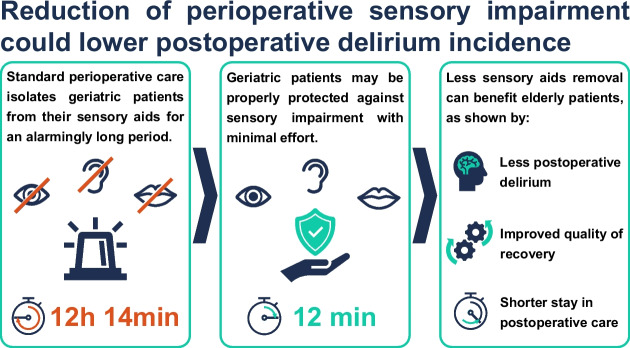
Visual abstract. A visual summary of the study’s key results

A decrease in the perioperative duration of sensory impairment, which means the time patients are without their required sensory aid devices, is related to a lower incidence of POD. Even though multimodal delirium prevention programs have been shown with high evidence to be effective against POD in a geriatric population, implementation of these programs requires substantial resources [15, 16]. With the results of this prospective cohort study, we demonstrate that (a) perioperative patients are separated from their sensory aid devices for an alarmingly long time in usual care, (b) the time of sensory impairment can be shortened very effectively with little effort, and (c) patients profit from these efforts by suffering from less postoperative delirium, showing better early quality of recovery, and requiring less postoperative monitoring time (Fig. 7). Supplying perioperative patients with their sensory aid devices as soon as possible may be a crucial part of a multimodal prevention program and should be implemented even if resources are limited. Especially given that POD relates to substantial financial damages and severe long-term outcomes for patients [8–10].

Sensory impairment is a highly prevalent condition in the elderly. The sensory impairment that results from withholding sensory aid devices from these patients is a well-known risk factor for POD [1, 17, 18]. Depending on age group, between 54.6% and 90.2% of individuals suffer from hearing loss, and between 16.5% and 29.9% report moderate to severe visual impairment [18, 34]. Most geriatric trauma patients in our research were admitted to the emergency department with SAD. Nevertheless, one in seven patients admitted to the emergency department via emergency services arrived there with their SAD left at home. There may be a lack of awareness regarding the importance of SAD among emergency responders that needs to be addressed. To prevent liability for lost patients' property and complications caused by SAD (e.g., ingestion of dentures), many hospitals prefer the safe storage of SAD on the ward while the patient is undergoing surgery [35]. This research demonstrates that perioperative sensory impairment is significantly reduced with little effort by using SAD in the perioperative setting (Fig. 3). During the conduct of this study, no losses or write-offs were recorded, and no SAD was displaced in direct linkage to the utilization of the transport case. It only came to the loss of one transport box when it was mistakenly transferred with the patient and his SAD to a rehabilitation center.

The preservation of the patient’s ability to communicate with caregivers and interact with the environment is an essential component of POD prevention [36]. The provision of dentures improves the patient's capability to communicate and interact by improving voice clarity [37]. As a result of enhanced perception via hearing and visual aids, the patient’s capability to orient himself in his environment is increased [38, 39]. This is crucial since proximal femoral fracture and its subsequent emergency surgery are painful and disturbing experiences for older patients, which can be attenuated by respectful communication and careful explanation of all measures to be performed on the awake patient [40–42]. Withholding SAD results in subsequent sensory impairment, which results in the patient feeling disoriented and stressed. Since pain and anxiety relief involve communication, the absence of SAD makes efforts more complicated [7, 13, 43]. This mechanism is a likely explanation for the considerable decrease in POD incidence observed in the present study (Fig. 4). When studying the incidence of POD for each follow-up point separately, the incidence of POD between groups differed significantly only at follow-ups five and six. In follow-ups one to four, there was a mere trend toward a lower POD incidence in IC without reaching statistical significance (Table 3). These findings might be explained by improved sensory input and output in the early postoperative period, which prevents a POD with a delayed onset but not one that manifests promptly after anesthesia.

Reduced perioperative sensory impairment is associated with a reduced time of postoperative monitoring (PACU and ICU) (Fig. 6). SAD can prevent misunderstandings in communication, which facilitates assessment of the patient’s condition [44]. A wider use of SAD may consequently result in a more efficient use of ICU resources. The low financial expenses of the presented strategy to reduce perioperative sensory impairment by decreased sensory aid removal highlight these cost benefits, considering that ICU care costs around 1,425 euros per day [45].

Furthermore, the decrease in perioperative sensory impairment improved the median QoR-9 throughout the hospitalization. It is plausible that the availability of SAD increases quality of life and that missing dentures cause discomfort for the patient [46, 47]. Patients express improved well-being and are more satisfied with their hospital stay as a result (Fig. 5). In this small cohort study, no effect on functional recovery according to the Barthel Index or subjective recovery according to QoR-9 after one month could be observed. Nonetheless, a decrease in sensory aid removal enhanced patient satisfaction as well as subjective recovery during the postoperative period and reduced the time of postoperative care. In terms of the perception of health professionals of SAD utilization in the perioperative setting, further research is required to provide reliable evidence.

### Study limitations and strengths

The present study has some limitations. Inclusion of patients into the study occurred during the COVID-19 pandemic, a unique setting that may have contributed to a greater prevalence of complications, as reflected by a prevalence of POD above average in our study [5, 48]. Due to the before-and-after design of this study, IC and CC have been exposed to different phases of the pandemic, which could have led to systematic bias.

Regarding baseline characteristics, patients in CC were physically less resilient (MET < 4). This finding may raise suspicion that the higher incidence of POD in CC was due to patients being sicker rather than patients in IC profiting from the intervention. The difference in MET is an isolated finding that is not accompanied by other variables reflecting illness severity. It is important to be aware that there is an established association between preoperative frailty and POD [49]. Given the IC's superior Parker Mobility Score, which indicates inferior mobility, this aspect deserves to be properly accounted for when interpreting the results. There were no notable differences in the incidence of anemia, renal function, perioperative inflammation, Identification of Seniors at Risk Score, or Charlson Comorbidity Index between groups. MET assessment via interview has been reported to have uncertain predictability in elderly patients [46]. Nevertheless, it is important to practice proper care when interpreting the data provided. This is because any differences in outcomes seen may not be completely due to the intervention but might also be influenced by the initial health state of the groups being compared.

The before-and-after design of this study does not allow for a judgment on the effectiveness of sensory impairment reduction in POD prevention, nor is it a proof of concept. Rather, this exploratory investigation found an association between perioperative sensory aid removal and POD frequency. Despite carefully conducted study planning, propensity score matching, and other statistical approaches to compensate for non-randomization, the design of this study remains susceptible to unmeasured confounding. Unmeasured confounding may have occurred due to temporal changes in patient care during the study period and a lack of blinding of staff and researchers. Further limitations include the small sample size caused by an unanticipatedly high dropout rate. Larger studies, ideally randomized controlled trials, are necessary to determine the effectiveness of reducing sensory aid removal in reducing the incidence of POD.

However, this study has several strengths. This research is, to our best knowledge, the first investigation to quantify the prevalence of patients requiring SAD and the time of stay without the required devices perioperatively in geriatric trauma surgery. Furthermore, it revealed a lack of awareness for SAD among rescue service personnel, as one in seven patients were admitted to the emergency room without their SAD, even though almost every elderly patient possesses some [19]. Finally, we were able to demonstrate an association between perioperative sensory impairment and both the incidence of POD and subjective recovery.

Risk factors of level I and II-III hospitals in the national registry for geriatric trauma do not substantially vary from the baseline and treatment data of the present investigation [50]. Within the confines of the study’s design and limitations, this research’s findings therefore have a considerable degree of external validity. Moreover, as shown by our results, the current intervention can be implemented with great success in a large hospital with 1,500 beds. Consequently, the evaluated strategy is highly generalizable and feasible for hospitals of comparable size or smaller.

## Conclusion

Perioperative sensory impairment by removing sensory aid is an important risk factor for POD, which can be minimized with minimal effort. Decreased sensory impairment may reduce the incidence of POD, improve postoperative recovery, and reduce the time of postoperative care. Minimizing perioperative time without SAD should thus be seen as a crucial component of future preventive efforts. While our findings confirm the usefulness of our intervention, it should not preclude initiatives to deploy contemporary multicomponent delirium prevention programs. Minimizing perioperative sensory impairment is rather emphasized as an effective and crucial component in comprehensive preventative approaches to reduce the harm and discomfort caused by POD in many patients.

## Data Availability

The datasets used and/or analyzed during the current study are available from the corresponding author on reasonable request.

## Abbreviations

3D-CAM: 3-Minute Diagnostic Confusion Assessment Method
ASA-Classification: American Society of Anesthesiologists-Classification
CC: Control cohort
CI: Confidence interval
e.g.: For example (abbreviation of Latin “exempli gratia”)
Fig.: Figure
FU: Follow-up
IC: Intervention cohort
ICU: Intensive care unit
MET: Metabolic Equivalent of Task
MoCA: Montreal cognitive assessment
PACU: Post anesthesia care unit
POD: Postoperative delirium
PONV: Postoperative nausea and vomiting
QoR-9: Quality-of-recovery-9 score
SAD: Sensory aid device(s) (e.g. dentures, glasses and hearing aids)
vs.: Versus
